# Near coding-complete genome sequence of 12 dengue serotype 2 viruses from the 2023 outbreak in Bangladesh

**DOI:** 10.1128/mra.00162-24

**Published:** 2024-05-03

**Authors:** Md. Abdullah Omar Nasif, Saikt Rahman, Manjur Hossain Khan Jony, Mohammad Tanbir Habib, Murshida Khanam, Sharmin Sultana, Mahbubur Rahman, Ahmed Nawsher Alam, Firdausi Qadri, Tahmina Shirin

**Affiliations:** 1 Institute of Epidemiology, Disease Control and Research (IEDCR), Dhaka, Bangladesh; 2 Institute for Developing Science and Health Initiatives (ideSHi), Dhaka, Bangladesh; Katholieke Universiteit Leuven, Leuven, Belgium

**Keywords:** dengue, Bangladesh, 2023 outbreak, serotype 2, sequencing, genotype II

## Abstract

We report the near coding-complete genomes of 12 DENV serotype 2 strains collected during the 2023 dengue outbreak in Bangladesh. Analyses showed that all 12 strains were closely related and belonged to genotype II-Cosmopolitan.

## ANNOUNCEMENT

Dengue virus, a single-stranded positive-sense RNA virus belonging to *Flaviviridae* family within the *Orthoflavivirus* genus, is responsible for causing dengue (1). Bangladesh has been experiencing Dengue outbreaks every year since 2000. But the 2023 outbreak surpassed all previous records with 321,179 hospitalized cases including 1,705 deaths (2). In 2023, Dengue serotype 2 replaced the previously predominant Dengue serotype 3, which had been circulating since 2019 (3, 4).

The Institute of Epidemiology, Disease Control & Research, mandated by the government for outbreak investigation and response has gathered dengue NS1-positive serum samples from various laboratories for serotype surveillance using the CDC Real-Time RT-qPCR Assay (5) amidst the outbreak. For sequencing, 12 DENV2-positive samples with a C*_T_* <27 were selected (Table 1).

**TABLE 1:** Sequencing findings and other related information

Serial	Sequence ID	Specimen collection date (YYYY-MM-DD)	Geographical location	Sex	Age (Years)	Patient status during specimen collection	C_T_ value	SRA^a^ accession no	GenBank accession no.	Number of passed reads	Read length N50	Sequence length (bp) (Sequence start and end position)	Mean sequence depth (×)	GC content (%)
1	mi0001	8/20/2023	Cumilla	Male	42	unknown	18.6	SRR27795913	PP309840	16,295	1589	7472	2090	44
2	mi0002	8/5/2023	Dhaka	Male	40	Hospitalized	23	SRR27795912	PP309841	8,915	1621	(97–7568)	1211	44
3	mi0003	7/12/2023	Dhaka	Male	70	unknown	15.3	SRR27795910	PP309842	8,907	1604		1109	44
4	mi0004	9/1/2023	Dhaka	Male	52	Hospitalized	19.2	SRR27795909	PP309843	10,642	1569		1066	44
5	mi0005	9/2/2023	Dhaka	Male	7	Hospitalized	19.6	SRR27795908	PP309844	21,663	1587		2816	44
6	mi0006	9/9/2023	Dhaka	Female	3	Hospitalized	19.4	SRR27795907	PP309845	13,925	1583		1754	44
7	mi0007	7/22/2023	Dhaka	Female	58	Hospitalized	18.7	SRR27911978	PP309846	45,697	1592		5963	44
8	mi0008	9/16/2023	Dhaka	Male	45	Hospitalized	17.8	SRR27795906	PP309847	5,986	1559		744	44
9	mi0009	9/22/2023	Dhaka	Male	17	Hospitalized	15.3	SRR27795905	PP309848	7,721	1564		791	44
10	mi0010	8/21/2023	Dhaka	Female	40	Hospitalized	19.3	SRR27795904	PP309849	20,684	1578		2217	44
11	mi0011	11/16/2023	Dhaka	Male	20	Hospitalized	22.2	SRR27795903	PP309850	35,384	1576		3754	44
12	mi0012	11/18/2023	Dhaka	Female	22	Hospitalized	23.7	SRR27795911	PP325839	12,832	1570		1441	44

^*a*^ Sequence Reads Archive.

Viral RNA was extracted from 140 µL of serum using QIAamp Viral RNA mini Kit (QIAGEN, Germany), converted into first-strand cDNA using LunaScript RT SuperMix (New England Biolabs, USA) using the random priming strategy and amplified with Q5 Hot start high fidelity 2× master mix (New England Biolabs, USA) using the 10 sets of primers described by Christopher et al. (6). The primers produced a set of 10 overlapping amplicons, spanning lengths from 1.15 to 1.85 kb. This approach allowed the sequencing of the near coding-complete genome, except for a part of the 3′ end (including the NS5), and the 3′ UTR. The amplicons were pooled per sample, cleaned with AMPure XP beads (Beckman Coulter, USA) and were end-repaired with NEBNext Ultra II end repair/dA-tailing module (New England Biolabs, USA), followed by barcoding with EXP-NBD104 (Oxford Nanopore Technologies, UK). The barcoded amplicons were normalized, pooled, and cleaned with AMPure XP beads, followed by final Library preparation with Ligation Sequencing Kit LSK109 (Oxford Nanopore Technologies, UK) and sequenced in a standard flow cell FLO-MIN106 (version 9.4.1) for 24 h. MinKNOW v22.12.5 was used for base calling and demultiplexing of raw reads. The library generated 311,942 reads, with 243,036 reads successfully passing through the quality filters.

Read quality of fastq files was assessed with NanoPlot v1.42.0 (7) and filtered and trimmed using Chopper (7). Read Mapping and alignment were done with epi2me-labs/wf-alignment workflow v0.6.1 (8) using the NC_001474.2 as reference. BAM files were sorted, indexed with samtools v1.19.2, and variants were called with bcftools v1.19 (9). Draft fasta consensus sequences were generated with seqtk v1.3-r106 (10) and the final consensus fasta sequences were obtained after polishing with medaka v1.11.3 (11). The sequences exhibit >99% nucleotide similarity among themselves and >92% similarity with the reference strain NC_001474.2. The sequencing findings, and associated sample metadata were summarized in Table 1.

**Fig 1:**
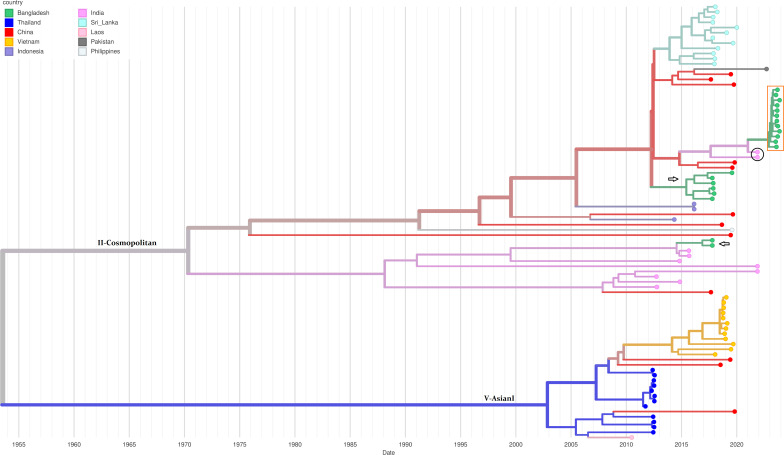
Time-resolved Phylogenetic tree of Dengue virus serotype 2 strains showing the 84 Asian genomes sampled between 2010 and 2023. The tree was built and annotated using Nextstrain tool augur. The tips were colored according to their location. The study strains were marked with the orange box and the closest Indian strains with black circle. The arrows indicated the previously circulating strains from Bangladesh. The date was represented in the unit of year.

A time-resolved phylogenetic tree (Fig. 1) of 84 Asian Dengue 2 viruses was built using Nextstrain tool augur and visualized using auspice (12). The tree showed the circulation of II-Cosmopolitan and V-AsianI genotypes. All 12 study strains were assigned to genotype II-Cosmopolitan and clustered closely together, with Indian strains as their closest relative. Characteristically, previously circulating Bangladeshi strains formed two separate lineages within the same clade and related distantly to the study strains.

## Data Availability

The data from this study can be found in GenBank under the accession number PP309840 to PP309850 and PP325839. The raw sequencing read were submitted at Sequence Read Archive (SRA) database under BioPorject PRJNA1071311 with the individual accession number starting from SRR27795903 to SRR27795913 and SRR27911978.
